# Clinical significance of miR-1180-3p in hepatocellular carcinoma: a study based on bioinformatics analysis and RT-qPCR validation

**DOI:** 10.1038/s41598-020-68450-z

**Published:** 2020-07-14

**Authors:** Zihan Zhou, Xianguo Zhou, Yanji Jiang, Moqin Qiu, Xiumei Liang, Qiuling Lin, Qian Guo, Cunli Nong, Rongrui Huo, Qian Chen, Haizhou Liu, Yingchun Liu, Shaoliang Zhu, Mengyun Wang, Hongping Yu

**Affiliations:** 1 Guangxi Medical University Cancer Hospital, Nanning, Guangxi China; 2 School of Public Health, Guangxi Medical University, Nanning, Guangxi China; 3 Department of Infectious Disease, Liuzhou Workers’ Hospital, Liuzhou, Guangxi China; 4 Fudan University Shanghai Cancer Center, Shanghai, China

**Keywords:** Cancer, Biomarkers, Cancer

## Abstract

miRNAs play an indispensable role in human carcinogenesis. Dysregulated miR-1180-3p has been observed in several types of cancer, including hepatocellular carcinoma (HCC). This study intends to correlate the expression level of miR-1180-3p with clinical features and overall survival in HCC patients. The expression and clinical significance of miR-1180-3p, selected from GEO and TCGA databases, were verified using an RT-qPCR method. The target genes of miR-1180-3p were obtained using 3 miRNA target gene prediction databases, and their functions were analyzed using the online tool WebGestalt. miR-1180-3p expression was significantly upregulated in 88 HCC tissues compared with non-tumor liver tissues (0.004 ± 0.009 vs. 0.002 ± 0.002, t = − 2.099, *P* = 0.038). Additionally, we found that the expression levels of miR-1180-3p were significantly correlated with tumor number (χ^2^ = 9.157, *P* = 0.006) and MVI (χ^2^ = 11.354, *P* = 0.003). Based on Kaplan–Meier analysis, patients with high miR-1180 expression had a shorter overall survival than those with low miR-1180-3p expression (*P* = 0.002). Furthermore, multivariate Cox analyses indicated that miR-1180-3p expression was an independent prognostic factor for overall survival (HR = 13.36, 95% CI 1.16, 153.69, *P* = 0.038). In addition, a total of 733 target genes of miR-1180-3p were found from three prediction databases. The GO analyses demonstrated that the target genes were closely related to the proliferation and malignancy of tumors. The KEGG analysis showed that target genes were enriched in several key cancer-related signaling pathways, including the Pathways in cancer, the Ras signaling pathway, and the MAPK signaling pathway. In conclusion, we demonstrate that miR-1180-3p is upregulated in HCC and is associated with a poor prognosis. Thus, miR-1180-3p might be useful as a prognostic marker for HCC.

## Introduction

Primary hepatic carcinoma is one of the most frequently occurring malignant tumors in the world. It is estimated that there are approximately 841,000 new cases and 782,000 deaths worldwide each year^1,2^. Hepatocellular carcinoma (HCC), the most common type of primary hepatic carcinoma, makes up 75% to 85% of all the liver cancers burden worldwide. Previous research demonstrated that numerous genetic and environmental risk factors, including HBV/HCV infection, smoking status and drinking status, play leading roles in HCC development^3,4^. At present, there are no effective treatments or early diagnostic makers for HCC patients, resulting in an extremely poor prognosis in HCC with a 5-year survival rate of less than 15%^5,6^. Thus, identification of novel well-performing diagnostic and prognostic biomarkers for HCC is urgently needed.

MicroRNA (miRNA) is a type of short non-coding RNA that is well-conserved. It can bind to the 3′-untranslated regions (3′-UTRs) of the target mRNA, leading to degradation of the target mRNA and inhibition of protein expression^7–9^. Mounting evidence indicates that miRNAs play a critical role in tumorigenesis^10–12^ by participating in a series of biological processes, involving apoptosis, migration and cell proliferation^13,14^. Indeed, the dysregulation of various miRNAs has been identified in malignant tumors and may serve as prognostic biomarkers and therapeutic targets^15–17^. In humans, miR-1180-3p is expressed from chromosome 17 (p11.2), and this miRNA may play different roles in carcinogenesis as drivers, oncogenes and tumor suppressors^18–20^. For example, Zhu et al.^19^ found that plasma miR-1180-3p is downregulated in early gastric cancer, suggesting that plasma miR-1180-3p can be used as new potential biomarker for the diagnosis of early gastric cancer. Simon et al.^20^ reported that miR-1180-3p upregulation in adenoid cystic carcinoma and was associated with improved recurrence-free survival.

In this study, we screened differentially expressed miRNAs in HCC using the GSE36915 dataset from GEO database, and three significantly overexpressed miRNAs were identified in HCC tissues. We further analyzed the relationship between three upregulation miRNA and the prognosis of HCC using the TCGA database, and found high expression levels of miR-1180-3p were correlated closely with poor outcomes of HCC patients. Hence, we validated miR-1180-3p expression using 88 pairs of HCC tissues and matched non-tumor liver tissues and investigated the clinical significance of miR-1180-3p in HCC. Furthermore, to thoroughly understand the biological function and pathways of miR-1180-3p, we also predicted the miR-1180-3p target genes, followed by functional enrichment analysis.

## Materials and methods

### The GEO database

The GSE36915 dataset (https://www.ncbi.nlm.nih.gov/geo/query/acc.cgi?acc=GSE36915), which contains the miRNA expression profiles from 68 HCC tissues and 21 non-tumor liver tissues, was downloaded from GEO.

### Screening of abnormal expression miRNAs

The “limma” package in R was used to define the abnormally expressed miRNAs in 68 HCC tissues and 21 non-tumor liver tissues^21^; miRNAs with a false discovery rate (FDR) < 0.001 and |Log_2_FoldChange (FC)|≥ 1.2 were considered abnormally expressed. The “ggplot2”^22^ package was used to visualize the abnormally expressed miRNAs in HCC and non-tumor liver.

### TCGA database

We downloaded the publicly available miRNA-Seq and survival data from TCGA (https://portal.gdc.cancer.gov/). We obtained the miRNA profiles of 371 HCC and 50 non-tumor liver samples together with the corresponding prognosis information. The “DESeq2”^23^ package was used to normalize and standardize the miRNA expression data from TCGA^24^.

### Screening of prognostic miRNAs

Based on survival data from TCGA, patients with follow-up times of less than 30 days were excluded. Ultimately, a total of 343 HCC patients were enrolled in this study for survival analysis. The association between miRNA expression and overall survival in HCC was analyzed using the Kaplan–Meier method and Cox proportional hazards regression models.

### Patient and tissue samples

Eighty-eight patients with HCC in our study underwent liver cancer resection at Guangxi Medical University Cancer Hospital from January 2017 to March 2018. No patients received treatment before surgery^10^. All HCC patients were reviewed to analyze clinical features, including age, gender, smoking status, drinking status, HBV/HCV infection, Edmonson grade, tumor number, largest tumor size, microvascular invasion (MVI), cirrhosis, and satellite nodule presence. Individuals who had smoked more than 6 months continuously or cumulatively in their lifetimes were defined as “ever smokers” and the rest as “never smokers. Those subjects who had drunk alcoholic beverages at least once a week for more than 6 months were defined as “ever drinkers” and the rest as “never drinkers”. Patients who were hepatitis B surface antigen (HBsAg) positive were defined as HBV infection, and who were hepatitis C antibody positive were defined as HCV infection. MVI was defined as the invasion of tumor cells (more than 50 invading tumor cells) in intrahepatic portal vein or hepatic vein branches. We divided the patients into three group: non-MVI group, mild MVI group (number of invaded micro vessels ≤ 5) and severe MVI group (number of invaded micro vessels > 5). This study was approved by the Ethics Committee of the Guangxi Medical University Cancer Hospital and was conducted in accordance with the principles of the Declaration of Helsinki. All participants voluntarily agreed to participate in this study and all gave written informed consent.

### RNA extraction and quantitative real-time PCR

TRIzol reagent (Invitrogen, Life Technologies) was used to isolate total RNA, and a Thermo Scientific Nanodrop 2000 was used to assess the RNA quality. For qPCR analysis, a Mir-X miRNA First-Strand Synthesis Kit (Clontech, CA, USA) was used to perform reverse transcription of total RNA. The TB Green Premix Ex Taq II kit (TaKaRa Bio, Shiga, Japan) was used according to the manufacturer’s instructions, and qPCR was performed using a Real-Time PCR Thermal Cycler. The reactions were as follows: 95 °C for 30 s, followed by 40 cycles of 95 °C for 5 s and 60 °C for 30 s. Each sample was assayed in triplicate. The relative expression level of miR-1180-3p was calculated using the comparative Ct method with the 2^−ΔCt^ formula^10^. The sequences of the miR-1180-3p and U6 primers are listed in Table 1.

**Table 1:** The sequences of the primers in this study.

Primers	Sequences
**miR-1180-3p**	
Forward	5′-CAGAAACAGCCATCCCAGAG-3′
Reverse	5′-GCCTTCAGCAGGATGTCAAT-3′
**U6**	
Forward	5′-CTCGCTTCGGCAGCACA-3′
Reverse	5′-AACGCTTCACGAATTTGCGT-3′

### Functional and pathway analysis

TargetScan, miRTarBase and miRDB databases were used to identify the target genes of miR-1180-3p, and the WEB-based GEne SeT AnaLysis Toolkit (WebGestalt)^25^ was used to perform Gene Ontology (GO) enrichment analysis and Kyoto Encyclopedia of Genes and Genomes (KEGG) pathway analysis.

**Table 2:** Cox proportional hazards regression analysis of 3 upregulated miRNAs affecting overall survival of HCC patients in TCGA.

	Univariate Cox analysis		Multivariate Cox analysis^a^	
	HR (95% Cl)	*P*	HR (95% Cl)	*P*
miR-183-5p	1.09(1.01,1.17)	0.035	1.06(0.98,1.14)	0.164
miR-452-5p	1.11(0.99,1.25)	0.073	\	\
miR-1180-3p	1.28(1.10,1.47)	0.001	1.25(1.07,1.45)	0.004

^a^Adjusted for the expression of miR-1180-3p and miR-183-5p.

### Statistical analysis

Continuous variables were shown as the mean ± SD and analyzed by student t test. The different expression profiles of miR-1180-3p between HCC tissue and non-tumor liver tissue were analyzed by T test, and the differences in clinical features between the low and high miR-1180-3p expression groups were evaluated by Chi-square test. For survival analysis, the Kaplan–Meier method was applied for univariate survival analysis, and Cox proportional hazards regression was conducted for multivariate survival analysis. The optimal cutoff values of miRNA expression levels were determined by the “surv_cutpoint” function of the “survminer” R package, and the cutoff values of miRNAs were used to classify patients with HCC into two groups (high expression or low expression)^26^. All the statistical analyses were performed utilizing R 3.5.2 software, and the results were considered to be significant at a *P* value less than 0.05.

## Results

### Screening of abnormally expressed miRNAs in HCC patients by bioinformatics analysis

**Figure 1:**
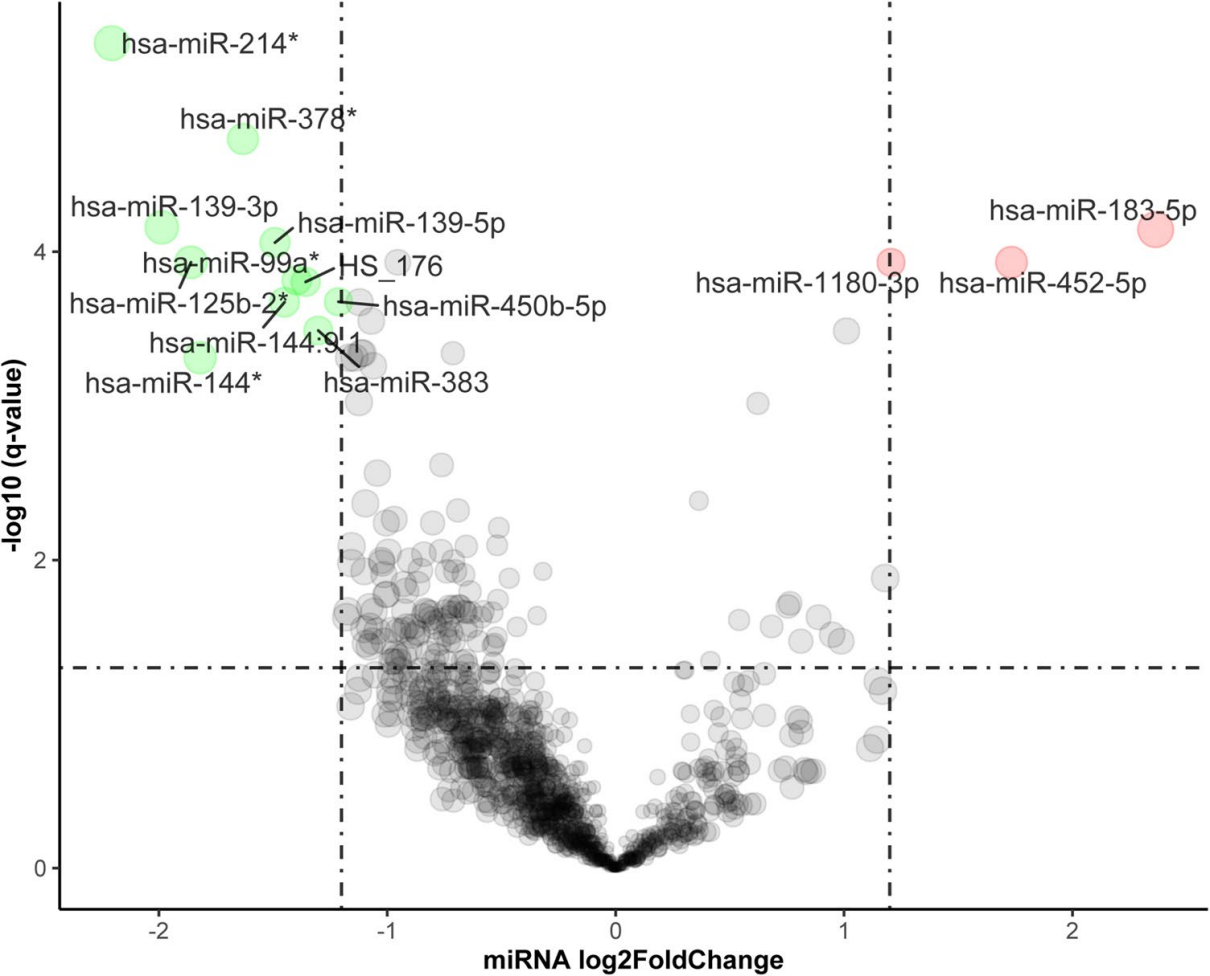
The different expression of miRNAs (GSE36915). The “limma” package in R (version 3.5.2) was used to define the abnormally expressed miRNAs and the volcano plot was created by “ggplot2” package. Red spots represent up-regulated genes, and green spots represent down-regulated genes.

We aimed to screen the abnormally expressed miRNAs in HCC. 14 significantly differentially expressed miRNAs (|Log_2_FC|≥ 1.2 and FDR < 0.001), including 3 upregulated miRNAs and 11 downregulated miRNAs, were identified in the GSE36915 dataset (Fig. 1). These results indicated that miR-183-5p (log_2_FC = 2.36, *P* < 0.001), miR-452-3p (log_2_FC = 1.73, *P* < 0.001), and miR-1180-3p (log_2_FC = 1.21, *P* < 0.001) may act as oncomiRs in HCC. Thus, these miRNAs were selected to further analysis.

### miR-1180-3p expression is related to the survival of HCC patients

**Figure 2:**
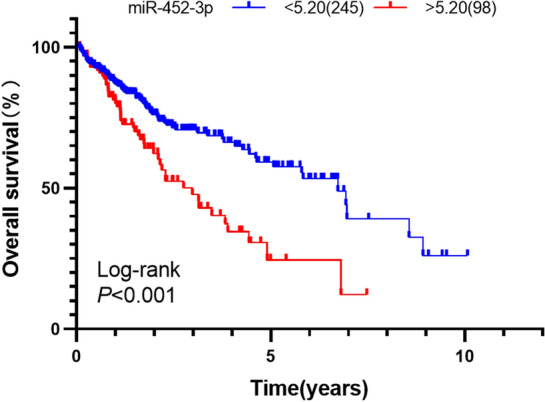
The correlation of miR-452-5p expression with overall survival of HCC patients in TCGA (Kaplan–Meier analysis). The survival curve was visualized by GraphPad Prism 8.0.2 (www.graphpad.com ).The cutoff values of miR-452-5p (cutoff = 13.45) was used to classify patients with HCC into high (n = 98) or low (n = 245) expression groups.

**Figure 3:**
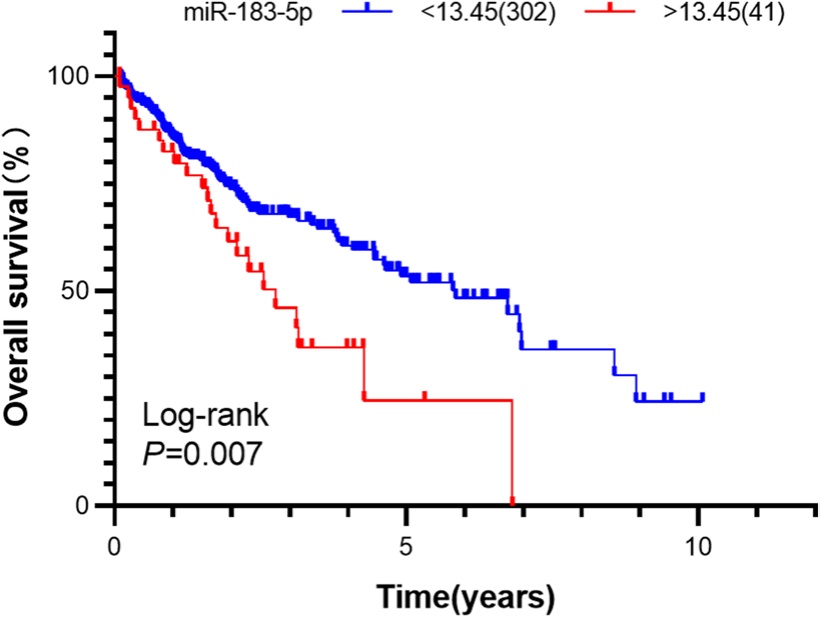
The correlation of miR-183-5p expression with overall survival of HCC patients in TCGA (Kaplan–Meier analysis). The survival curve was visualized by GraphPad Prism 8.0.2 (www.graphpad.com). The cutoff values of miR-183-5p (cutoff = 5.20) was used to classify patients with HCC into high (n = 41) or low (n = 302) expression groups.

**Figure 4:**
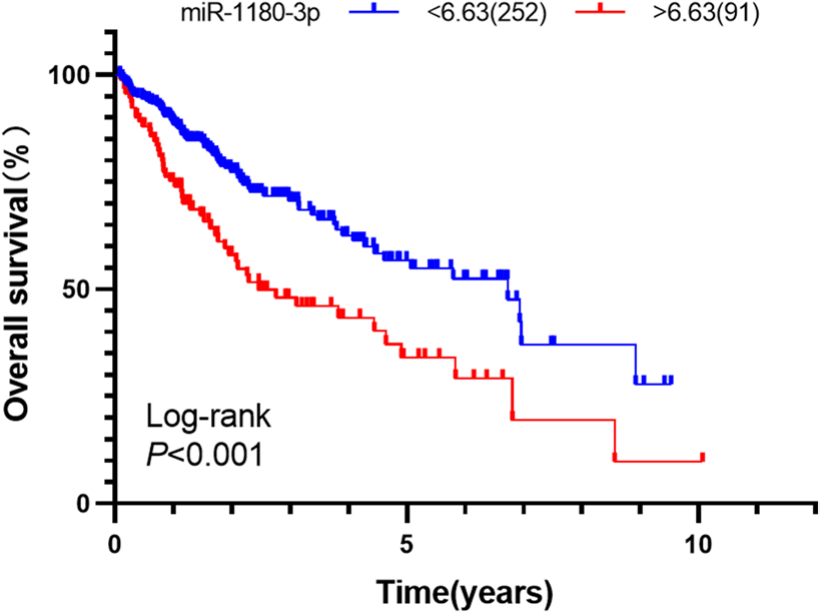
The correlation of miR-1180-3p expression with overall survival of HCC patients in TCGA (Kaplan–Meier analysis). The survival curve was visualized by GraphPad Prism 8.0.2 (www.graphpad.com). The cutoff values of miR-1180-3p (cutoff = 6.63) was used to classify patients with HCC into high (n = 91) or low (n = 252) expression groups.

To identify miRNAs related to the survival of HCC patients, we used TCGA datasets to correlate the expression of 3 upregulated miRNAs with overall survival in HCC patients (Figs. 2, 3, 4). Only miR-183-5p and miR-1180-3p were found to be related to the survival of HCC patients by both Kaplan–Meier survival analysis and univariate Cox analysis (Table 2). Patients with high expression of miR-183-5p and miR-1180-3p had short overall survival. Subsequently, miR-183-5p and miR-1180-3p were input into the multivariate Cox hazard model to test their independent impact on HCC, and the results showed that the expression of miR-1180-3p (HR = 1.25, 95%CI = 1.07–1.45, *P* = 0.004) was an independent prognostic factor for HCC (Table 2). Thus, miR-1180-3p was selected for in-depth investigation.

### miR-1180-3p is upregulated in HCC samples

**Figure 5:**
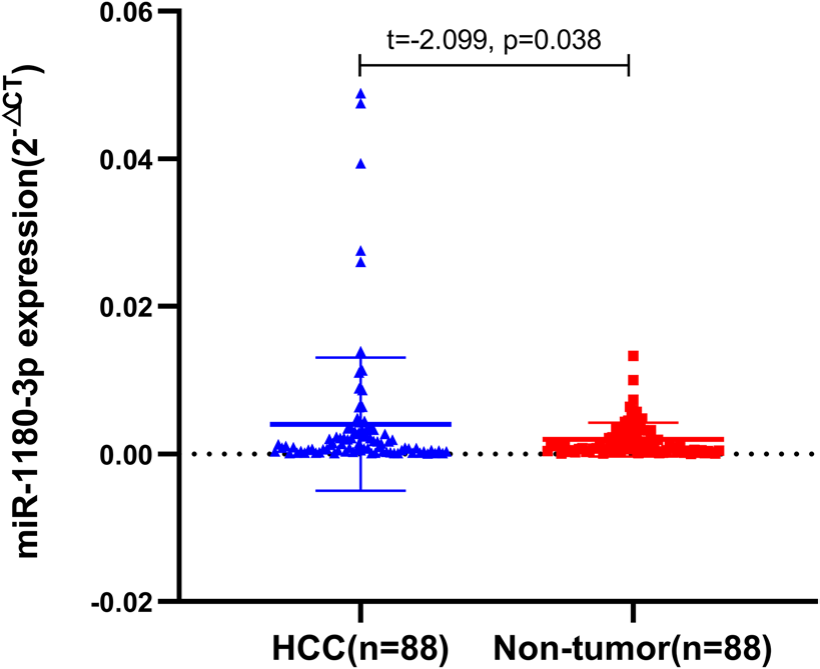
The expression of miR-1180-3p in HCC samples. The scatter diagram was created by GraphPad Prism 8.0.2 (www.graphpad.com). miR-1180-3p expression is significantly higher in HCC tumor tissues compared to matched Non-tumor liver tissues (0.004 ± 0.009 vs 0.002 ± 0.002, t = − 2.099, *P* = 0.038).

Based on bioinformatics analysis, miR-1180-3p was demonstrated to be highly expressed in HCC tissues. To validate this finding, we analyzed miR-1180-3p expression levels in 88 paired HCC tissue and non-tumor liver tissue by RT-qPCR. The results showed that, when compared with non-tumor liver tissue, the expression levels of miR-1180-3p were upregulated in HCC tissue samples (0.004 ± 0.009 vs. 0.002 ± 0.002, t = − 2.099, *P* = 0.038; Fig. 5).

### Correlation of miR-1180-3p with HCC clinical features

The correlations of miR-1180-3p with HCC clinical features were analyzed to clarify the roles of miR-1180-3p in the development of HCC, whereby patients were divided into two groups according to the cutoff value of miR-1180-3p (cutoff = 0.01). The results showed that miR-1180-3p expression level was significantly correlated with tumor number (χ^2^ = 9.157, *P* = 0.006) and MVI (χ^2^ = 11.354, *P* = 0.003). miR-1180-3p was overexpressed in samples with multiple tumors and severe MVI (Table 3). Furthermore, we noticed that, compared with patients with HCV infection negative, low Edmonson grade (I–II) and non-cirrhosis, patients with HCV infection positive, middle(II–III)/advanced (III–IV) Edmonson grade or cirrhosis appeared to have a higher proportion of high expression miR-1180-3p (25.0% vs. 11.9%, 13.1% vs. 11.1% and 16.3% vs. 7.7%, respectively), but the difference was not statistically significant (all *P* > 0.05, Table 3).

**Table 3:** Relationship between the expression of miR-1180-3p and clinicopathological characteristics in 88 HCC patients.

	miR-1180-3p expression, n (%)		χ^2^	*P*
	Low (n = 77)	High (n = 11)		
**Age(years)**				
≤ 54	41(87.2)	6(12.8)	0.007	0.936
>54	36(87.8)	5(12.2)		
**Gender**				
Female	14(77.8)	4(22.2)	1.956	0.225
Male	63(90)	7(10)		
**Smoking status**				
Never	50(84.7)	9(15.3)	1.242	0.326
Ever	27(93.1)	2(6.9)		
**Drinking status**				
Never	53(84.1)	10(15.9)	2.307	0.168
Ever	24(96)	1(4)		
**HBV infection**				
Negative	11(84.6)	2(15.4)	0.116	0.663
Positive	66(88.0)	9(12.0)		
**HCV infection**				
Negative	74(88.1)	10(11.9)	0.599	0.439
Positive	3(75.0)	1(25.0)		
**Edmonson grade**				
Low (I–II)	24(88.9)	3(11.1)	0.069	0.793
Middle(II–III)/Advanced (III–IV)	53(86.9)	8(13.1)		
**Tumor number**				
1	61(93.8)	4(6.2)	9.157	**0.006**
≥ 2	16(69.6)	7(30.4)		
**Size of largest tumor (cm)**				
≤ 5	25(86.2)	4(13.8)	0.066	1.000
>5	52(88.1)	7(11.9)		
**Microvascular invasion (MVI)**				
None	49(90.7)	5(9.3)	11.354	**0.003**
Mild (MVI ≤ 5)	24(92.3)	2(7.7)		
Severe (MVI > 5)	4(50.0)	4(50.0)		
**Cirrhosis**				
No	36(92.3)	3(7.7)	1.480	0.333
Yes	41(83.7)	8(16.3)		
**Satellite nodule presence**				
No	69(87.3)	10(12.7)	0.018	1.000
Yes	8(88.9)	1(11.1)		

Bold font indicates statistical significance (*P* < 0.05).

### miR-1180-3p is an independent prognostic factor for HCC

**Figure 6:**
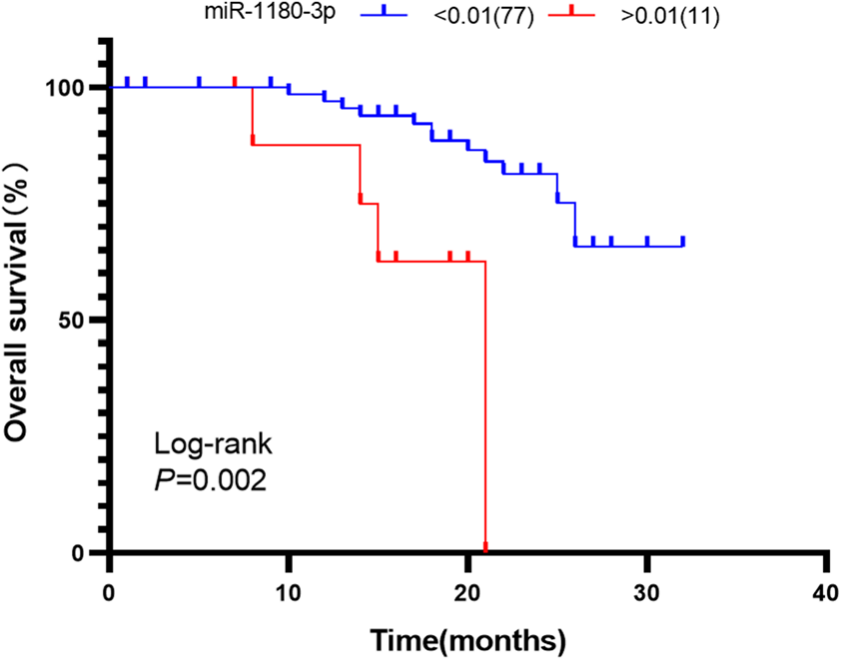
The prognostic significance of miR-1180-3p in HCC samples. The survival curve was visualized by GraphPad Prism 8.0.2 (www.graphpad.com). The log-rank test shows that HCC patients with high miR-1180-3p expression have higher overall survival than those with low expression of miR-1180-3p. The cutoff values of miR-1180-3p (cutoff = 0.01) was used to classify patients with HCC into high (n = 11) or low (n = 77) expression groups.

To identify the risk factors of prognosis in HCC patients, we analyzed the expression of miR-1180-3p and its clinical significance in HCC based on Kaplan–Meier models and Cox proportional hazards regression models. The log-rank test results showed that patients with high miR-1180-3p expression had a shorter overall survival than patients with low miR-1180-3p expression in HCC (*P* = 0.002; Fig. 6). Through univariate analysis, the expression of miR-1180-3p, tumor number, MVI and satellite nodule presence were significantly associated with overall survival in HCC patients (*P* < 0.05). Although age, gender, smoking status, drinking status, HBV infection, HCV infection, largest tumor size, and cirrhosis were not significantly related to the expression of miR-1180-3p or the prognosis of HCC, we still professionally thought them can affect the relationship between miR-1180-3p and HCC prognosis. Therefore, we inputted all clinical features in multivariate Cox analysis, and found that miR-1180-3p expression was defined as an independent prognostic factor for overall survival in patients with HCC (HR = 13.36, 95% CI: 1.16, 153.69, *P* = 0.038; Table 4).

**Table 4:** Cox proportional hazards regression analysis on the relationship of clinicopathologic characteristics and prognosis in 88 HCC patients.

	No. of death/patient	Mean OS (months)	Univariate Cox analysis		Multivariate Cox analysis^a^	
			HR (95%Cl)	*P*	HR (95%Cl)	*P*
**Age(years)**						
≤ 54	11/47	26.57	1			
>54	5/41	27.57	0.40(0.14,1.16)	0.091		
**Gender**						
Female	3/18	22.69	1			
Male	13/70	27.64	0.83(0.23,2.96)	0.769		
**Smoking status**						
Never	9/59	24.62	1			
Ever	7/29	26.59	1.22(0.45,3.31)	0.690		
**Drinking status**						
Never	8/63	27.37	1			
Ever	8/25	25.23	1.96(0.73,5.23)	0.180		
**HBV infection**						
Negative	2/13	22.18	1			
Positive	14/75	27.59	0.717(0.160,3.207)	0.663		
**HCV infection**						
Negative	16/84	17.94	1			
Positive	0/4	13.75	0.05(0.00–23,477.97)	0.648		
**Edmonson grade**						
Low	3/27	29.92	1			
Middle/Advanced	13/61	25.47	2.31(0.66,8.15)	0.191		
**Tumor number**						
1	7/65	27.58	1		1	
≥ 2	9/23	24.30	3.32(1.24,8.92)	0.017	1.42(0.35,5.74)	0.620
**Size of largest tumor (cm)**						
≤ 5	3/29	25.52	1			
>5	13/59	26.66	2.16(0.61,7.60)	0.230		
**Microvascular invasion (MVI)**						
None	6/54	29.46	1		1	
Mild (MVI ≤ 5)	5/26	22.69	2.34(0.68,8.11)	0.180	2.34(0.49,11.25)	0.290
Severe (MVI > 5)	5/8	15.92	14.61(4.19,50.96)	< 0.001	**7.39(1.03,53.09)**	**0.047**
**Cirrhosis**						
No	6/39	26.49	1			
Yes	10/49	27.25	1.11(0.40,3.06)	0.844		
**Satellite nodule presence**						
No	12/79	28.29	1		1	
Yes	4/9	18.13	6.19(1.90,20.21)	0.003	**25.87(3.38,199.90)**	**0.002**
**miR-1180-3p expression**						
Low	12/77	28.25	1		**1**	
High	4/11	17.75	5.52(1.66,18.33)	0.005	**13.36(1.16,153.69)**	**0.038**

*CI* confidence interval, *HR* hazard ratio, *OS* overall survival. ^a^Multivariate Cox regression analyses were adjusted for age, gender, smoking status, drinking status, HBV infection, HCV infection, Edmonson grade, tumor number, largest tumor size, MVI, cirrhosis, satellite nodule presence and miR-1180-3p expression. Bold font indicates statistical significance (*P* < 0.05).

### Functional and pathway analysis of miR-1180-3p

**Figure 7:**
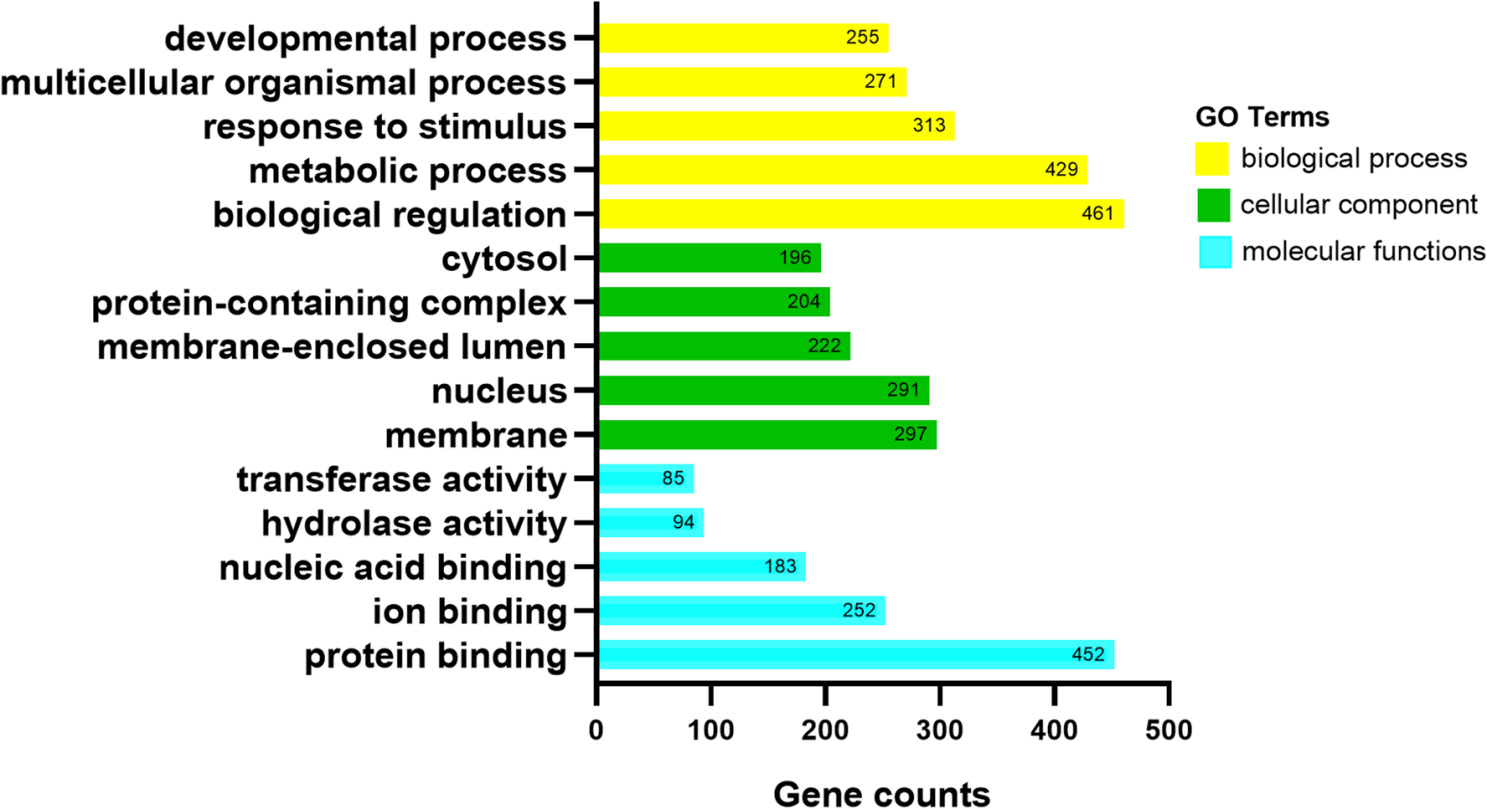
The GO analysis of miR-1180-3p target genes. WebGestalt (https://www.webgestalt.org) was used to perform Gene GO enrichment analysis and GraphPad Prism 8.0.2 was used to visualize results by created a histogram. The top 5 GO enrichment terms of target genes in biological process ontology, cellular component ontology, molecular function ontology.

**Figure 8:**
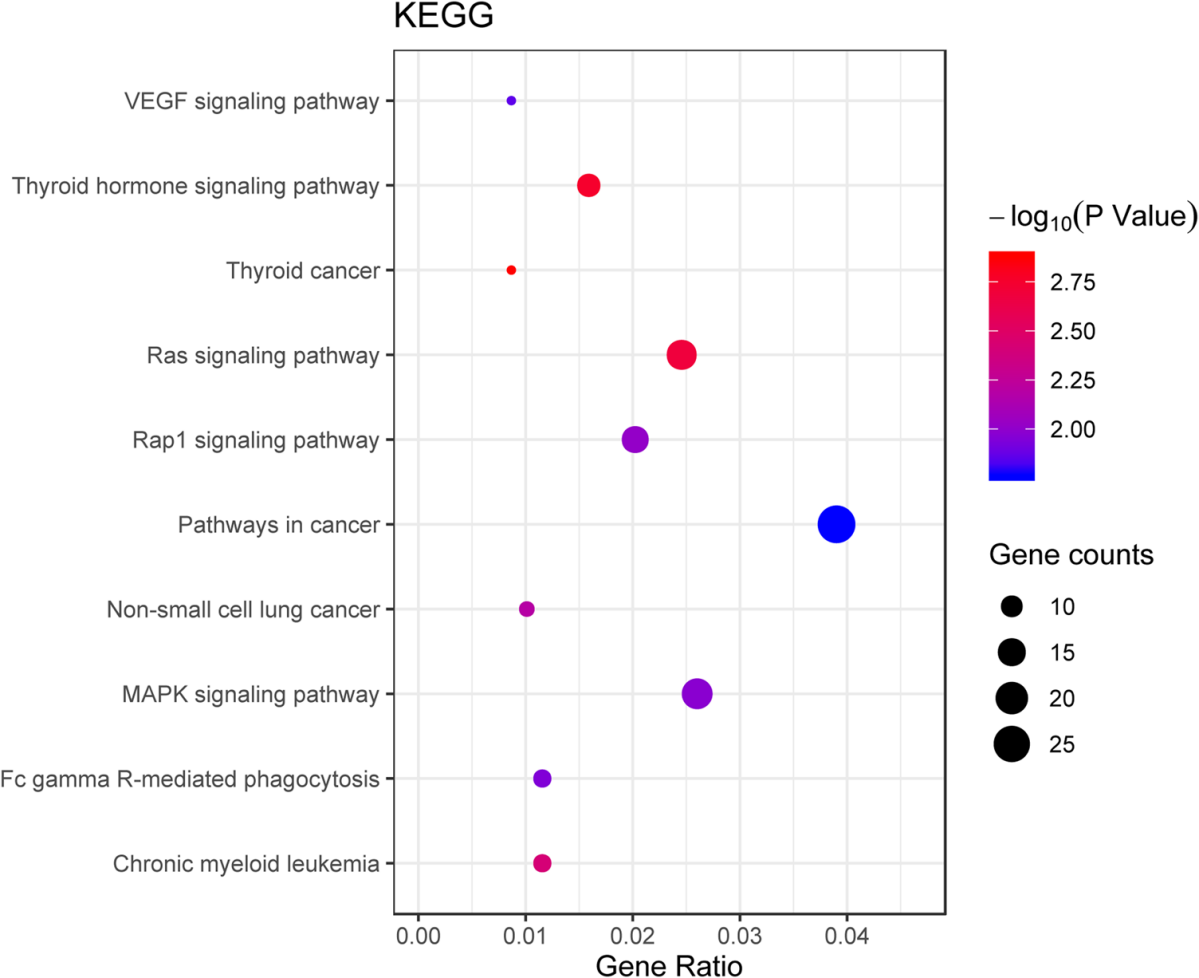
The KEGG analysis of miR-1180-3p target genes. WebGestalt (https://www.webgestalt.org) was used to perform KEGG analysis and Bubble chart was created by ggplot2 package in R 3.5.2. The top 10 signaling pathways of the miR-1180-3p target genes by KEGG analysis.

To further understand the potential mechanism of miR-1180-3p in HCC, we applied GO and KEGG analyses on the target genes of miR-1180-3p. A total of 733 target genes were found, of which 37 were annotated in miRDB, 683 in TargetScan and 67 in miRTarBase. The GO analysis results of the miR-1180-3p target genes indicated that, for biological process ontology, the target genes were mainly enriched in biological regulation, metabolic process and response to stimulus; for cellular component ontology, the target genes were particularly enriched in membrane, nucleus or membrane-enclosed lumen; for molecular functions, these target genes were involved in protein binding, ion binding and nucleic acid binding (Fig. 7). The top 10 KEGG pathways of the miR-1180-3p target genes by KEGG analysis are shown in Fig. 8. These results demonstrated that the miR-1180-3p target genes mainly participate in the Pathways in cancer, the MAPK signaling pathway and the Ras signaling pathway. Notably, the MAPK1, AKT1 and PRKCA genes were enriched in a vast majority of vital cancer-related signaling pathways (Table 5).

**Table 5:** The top 10 KEGG pathways of the miR-1180-3p target genes.

Description	Enrichment Ratio	p Value	Gene
Thyroid cancer	4.813	0.001	TPR;NTRK1;NCOA4;BAK1;RXRA;MAPK1
Thyroid hormone signaling pathway	2.814	0.002	ACTG1;BAD;MED13L;ATP1A1;CASP9;PLCE1;PRKCA;RXRA;MAPK1;AKT1;HDAC2
Ras signaling pathway	2.175	0.002	ETS1;CSF1;NTRK1;VEGFB;BAD;EFNA2;RASA4B;FGFR3;PLA2G4F;RASA4;PLCE1;PRKCA;MAPK1;AKT1;RASAL2;ANGPT4;GRIN2A
Chronic myeloid leukemia	3.124	0.004	BAD;BCR;CTBP1;BAK1;CBL;MAPK1;AKT1;HDAC2
Non-small cell lung cancer	3.148	0.006	BAD;CASP9;BAK1;PRKCA;RXRA;MAPK1;AKT1
Rap1 signaling pathway	2.017	0.01	CSF1;ACTG1;VEGFB;EFNA2;RAPGEF6;FGFR3;RAPGEF2;PLCE1;PRKCA;MAPK1;RAPGEF1;AKT1;ANGPT4;GRIN2A
MAPK signaling pathway	1.811	0.011	MKNK2;CSF1;NTRK1;VEGFB;CACNB2;EFNA2;MAP2K7;RPS6KA1;FGFR3;RAPGEF2;PLA2G4F;FLNB;PRKCA;DUSP2;MAPK1;AKT1;CACNG2;ANGPT4
Fc gamma R-mediated phagocytosis	2.609	0.011	MYO10;PLA2G4F;INPPL1;WASF2;PRKCA;ARPC5L;MAPK1;AKT1
VEGF signaling pathway	3.018	0.014	BAD;CASP9;PLA2G4F;PRKCA;MAPK1;AKT1
Pathways in cancer	1.529	0.017	ETS1;TPR;NTRK1;VEGFB;BAD;FRAT1;NCOA4;FGFR3;ITGA6;ARNT2;CASP9;BCR;GLI2;CTBP1;BAK1;WNT8B;CBL;PRKCA;RXRA;MAPK1;WNT9B;SP1;AKT1;SUFU;HDAC2;TRAF1;LAMA4

## Discussion

Despite the evolution of cancer pharmacological treatments and therapeutic strategies for HCC, a significant fraction of patients experience death because of late diagnosis; therefore, new diagnostic and prognostic biomarkers are needed^27^. In recent years, with the advent of the era of big data and the advancement of high-throughput technologies, the TCGA and GEO databases have collected and publicly shared huge amounts of raw data for various cancer that can be used by researchers to perform various bioinformatics analyses and to provide important biomarkers for the diagnosis and treatment of tumors^28^.

In this study, the GSE36915 dataset was used to systematically screen the abnormally expressed miRNAs in HCC. Subsequently, TCGA datasets were used to identify prognosis-related miRNAs in HCC patients. Based on bioinformatics analysis, miR-1180-3p, an upregulated miRNA in HCC, acted as an independent prognostic factor in HCC. miR-1180-3p was then selected for exploration of its clinical significance in independent HCC samples. Using an RT-qPCR method, we found that miR-1180-3p was upregulated in HCC tissues compared with that in non-tumor liver tissue. Pascut et al.^29^ examined the expression levels of 13 miRNAs including miR-1180-3p in the serums of HCV-related HCC patients and non-HCC patients, and found that no difference existed in miR-1180-3p between the two groups. Pascut et al.′s study^29^ is different from ours in that their study examined miR-1180-3p in the serums of HCV-related HCC and non-HCC patients, whereas ours examined miR-1180-3p in the tissues of HCC patients (71 out of 88 infected by HBV, and only 4 infected by both HBV and HCV). Additionally, we analyzed the differences in clinical features between the low and high miR-1180-3p expression groups, and we found that miR-1180-3p was overexpressed in HCC samples with multiple tumors and severe MVI. Although there was no evidence of an association between miR-1180-3p expression level and Edmonson grade, largest tumor size and cirrhosis, higher proportion of high expression miR-1180-3p were found among patients with HCV infection positive, middle/advanced Edmonson grade or cirrhosis. These results suggested that miR-1180-3p high expression indicates the extent of malignancy in HCC. Previous studies have reported miR-1180 is overexpressed in Wilms’ tumor, and its expression has been positively correlated with histopathological type, NWTS stage and lymphatic metastasis^30^. These results suggest that miR-1180-3p can promotes the occurrence and development of tumor, including HCC. For survival analysis, the univariate Cox analyses showed that miR-1180-3p high expression, multiple tumors, severe MVI and satellite nodule presence were related to a shorter overall survival of HCC patients. Furthermore, miR-1180-3p expression was defined as an independent prognostic factor for overall survival in patients with HCC using multivariate Cox analysis. Similarly, Dou D et al.^31^ proposed miR-1180 high expression as a biomarker for poor prognosis of pancreatic adenocarcinoma. These results suggested that miR-1180-3p can be a useful prognostic marker for HCC patients after liver cancer resection.

Previous studies have shown that miR-1180 can promote HCC cell proliferation by downregulating TNIP2 expression and induce apoptosis resistance by activating the NF-κB signaling pathway^32,33^. It has been found that the increased miR-1180 expression levels can promote cell proliferation, migration, and invasion by activating biological pathways, including Wnt/β-catenin signaling pathways and PI3K/AKT signaling pathways, in lung cancer^34,35^. Moreover, by activating the SFRP1/Wnt signaling pathway^35^, miR-1180 upregulation may accelerate the proliferation and glycolysis of ovarian cancer cells, and miR-1180 overexpression can lead to a poor prognosis for survival among ovarian cancer patients^36,37^. To explore potential biological processes and pathways of miR-1180-3p in HCC, we identified 733 target genes of miR-1180-3p from three prediction databases for functional enrichment analysis. In the GO analysis, we found that these target genes play a crucial role in the proliferation and malignancy of tumors. KEGG analysis showed that miR-1180-3p target genes were enriched in several key cancer-related signaling pathways, including the Pathways in cancer, the Ras signaling pathway, and the MAPK signaling pathway. Notably, the target genes of miR-1180-3p, such as MAPK1, AKT1 and PRKCA, were involved in multiple signaling pathways, suggesting that miR-1180-3p can promote tumorigenesis though regulating the expression of target genes to activate the signaling pathways in HCC. However, little is known about the co-expression relationships between miR-1180-3p and these target genes. Therefore, further in-vitro and in-vivo assays of the potential biological functions of miR-1180-3p in those signaling pathways in HCC are essential to verify and illuminate the regulatory mechanisms of miR-1180-3p in HCC.

Taken together, our findings verified that miR-1180-3p functioned as a tumor promoter, and the progression and metastasis of HCC may be attributed to its upregulation in tumor tissues, suggesting that miR-1180-3p is a potential therapeutic target for HCC. Some limitations must be acknowledged for this study. First, these results may not be representative of the large patient population. Second, the follow-up time was not sufficiently long. Finally, further research on the mechanisms of miR-1180-3p in HCC progression is needed. Therefore, more studies with longer follow-up times should be performed to confirm our conclusions.

## Conclusions

In light of these findings, we demonstrate that miR-1180-3p overexpression in HCC is associated with poor prognosis. Thus, miR-1180-3p might be useful as a prognostic marker for HCC. Further studies are still needed to confirm our findings and to explore the potential mechanism underlying the role of miR-1180-3p in HCC.

### Ethics approval and consent to participate

Written informed consent was obtained from all participants, and this study was approved by the Ethics Committee of the Guangxi Medical University Cancer Hospital and was conducted in accordance with the principles of the Declaration of Helsinki.

## Data Availability

The datasets used during the present study are available from the corresponding author upon reasonable request.
